# Recommendations for Integrating Evidence-Based, Sustainable Diet Information into Nutrition Education

**DOI:** 10.3390/nu13114170

**Published:** 2021-11-21

**Authors:** Gemma E. Bastian, Danielle Buro, Debra M. Palmer-Keenan

**Affiliations:** 1 Department of Nutritional Sciences, Rutgers University, New Brunswick, NJ 08901, USA; g.e.bastian@rutgers.edu; 2 Division of Life Sciences, Rutgers University, New Brunswick, NJ 08901, USA; burodanielle@gmail.com

**Keywords:** nutrition education, food systems, sustainability, sustainable diets, food-related environmental impacts, climate change

## Abstract

The adoption of more sustainable diets (SD) has the capacity to meet the needs of individuals without compromising future generations’ abilities to do the same. Nutrition educators are ideal candidates for delivering SD education to consumers, yet evidence-based recommendations for the profession have not been crafted. The results of a thorough, narrative review of the literature performed in 2021 suggest there are five well-supported recommendations nutrition educators should consider incorporating in their work. They are (1) shift towards a plant-based diet, (2) mitigate food waste, (3) limit consumption of ultra-processed foods (UPF), (4) engage in local food systems, and (5) choose sustainable seafood. Each recommendation is discussed below in detail, to provide nutrition educators with a nuanced scope of the issue, after which suggestions for the inclusion of these recommendations, using an example of the authors’ experiences from the US Expanded Food and Nutrition Education Program (EFNEP), are provided.

## 1. Introduction

As global trends regarding climate, land and water use, and air pollution have worsened over the past century [1], the need to view systems through a sustainability lens is apparent. Derived from the Brundtland Commission’s definition of “sustainable development” in 1987 [2], the term “sustainable” has been more recently defined as “the capacity of being maintained over the long term and meeting the needs of the present without jeopardizing the ability to meet the needs of future generations [3]. As the Food and Agriculture Organization of the United Nations (FAO) outlined in a framework of sustainable food systems, the needs of the present and future include ending hunger and improving both food security and nutrition [4], which additionally align with the United Nations’ 2030 Sustainable Development Goals [5]. Yet, with documented evidence that malnutrition exists in “every country in the world” [6], and with many nations facing what has been called the “triple burden of malnutrition” from overweight, undernutrition, and micronutrient deficiencies [6], it is apparent that the current global food system needs improvement to become sustainable. Moreover, grave concerns have been raised as to how to equitably feed the human race in the near future [7], as the global population has been estimated to approach 10 billion by 2050 [8]. It is likely that this population growth will create further strain on food systems, such as increasingly frequent and extreme weather events from human-derived climate change, that will result in ominous challenges to global food supply chains [9]. As such, a commission of prominent scientists (the EAT–*Lancet* Commission) have called for a “Great Food Transformation”, i.e., a radical and multi-level change across the food system, aiming to safely and sustainably feed the world’s people by the year 2050 [10].

The EAT–*Lancet* Commission outlined the adoption of a “universal” diet that would “provide major health benefits” and also increase sustainability [10]. Sustainability, as it refers to food and nutrition, is often termed “sustainable diets” (SD). The Food and Agriculture Organization of the United Nations (FAO) has further defined SD as diets “with low environmental impacts which contribute to food and nutrition security and to [sic] healthy life for present and future generations [that are] protective and respectful of biodiversity and ecosystems, culturally acceptable, accessible, economically fair and affordable; nutritionally adequate, safe and healthy; while optimizing natural and human resources [11]”. Since then, many have attempted to characterize the composition of sustainable diets within the context of food-based dietary guidelines. By consensus, SD that meet these criteria include high amounts of plant-based foods (e.g., vegetables, fruits, seeds, nuts, legumes, and whole grain foods) and moderate-to-low amounts of animal-based foods (e.g., meat, poultry, seafood, eggs, and dairy) [10,12,13,14,15,16,17,18].

There have been calls to promote SD to consumers [19,20,21,22], in part through nutrition education [23,24]. Nutrition education has been defined as “any combination of educational strategies, accompanied by environmental supports, designed to facilitate voluntary adoption of food choices and other food- and nutrition-related behaviors conducive to health and well-being [25]”, and has been conducted in a broad array of settings, including schools, colleges and universities, community sites, and clinical sites, to both youth and adults [26]. Of note, the counseling done by dietitians with their patients (typically at clinical sites) should also be considered as “nutrition education” for the purposes of this paper, as this work presents an equally viable opportunity for the incorporation of SD messages in such a setting. Calls to adopt SD into practice are by no means a new trend in nutrition education. Gussow and Clancy, two nutrition educators from New York, first coined the term “sustainable diets” in a seminal 1986 article in the Journal for Nutrition Education and Behavior (erstwhile Journal for Nutrition Education) [27].

Similar to evidence-based medicine [28], effective nutrition education is reliant on a strong evidence base grounded in research [29,30]. However, due to the rapidly growing and evolving literature on SD, it may be challenging for nutrition educators to identify the most potentially impactful, evidence-based recommendations for inclusion in their educational offerings, as well as the means for doing so. This article reviews and synthesizes the most current, available evidence on SD, based on which the authors have identified priority nutrition education SD concepts and provided practical recommendations based on both the literature and the authors’ experiences.

## 2. Assumptions Made for This Review

Due to the breadth of the topic being discussed, the authors used three assumptions to guide this narrative literature review, and consequently, the development of the recommendations offered herein.

The first assumption was that consumer-focused, actionable SD recommendations should be provided for the purchase or acquisition of food, as well as regarding its preparation, consumption patterns, and storage. Of course, even 100% effective SD consumer education could not independently render the food system sustainable. As per the EAT–*Lancet* Commission’s “Great Food Transformation [10]”, change would be required at all levels of the food system, including agriculture, processing, distribution, retail, and food service. Many of these changes lie beyond the consumer’s control. For instance, certain agricultural practices, such as organic, biodynamic, or regenerative farming, have impacts on one or more sustainability factors, yet economic factors, particularly the lack of availability of these foods and their prices, prevent many consumers from being able to purchase or acquire foods from such agricultural practices in the food system’s current state. Due to this assumption, the recommendations made herein are limited to those that are broad enough for most consumers to act upon given the present-day food environment.

The second assumption was that it would be most prudent to focus on consumer strategies for those in higher-income countries, as they would have the greatest ability to make positive SD changes. In addition to their greater accessibility to affordable SD options, critics of the EAT–*Lancet* Commission’s report have argued that “global” recommendations do not reflect the reality of people living in lower- and middle-income countries (LMIC) [31,32]. For example, increasing meat intake in some low-income countries may increase food security and help ameliorate undernutrition [33,34]. Since SD consumer strategies likely differ between higher-income and lower-income countries, and limited work has been published regarding SD recommendations for LMIC [35], this paper has been restricted to SD strategies for residents of higher-income nations.

The third assumption, in accordance with the FAO SD definition, was that SD recommendations must be beneficial for both planetary and human health. This assumption is in good alignment with nutrition education aims since, as eloquently stated by Contento, a nutrition educator’s primary goal is promoting “health and well-being [25]”. Specific nutrient concerns related to the recommendations made in this work are discussed throughout, where appropriate.

## 3. Scientifically Supported, Sustainable Diet Education Recommendations

Since the authors sought to identify and summarize previously published literature with the intent of addressing the current lack of knowledge, and to open discussion regarding a new area of study, i.e., what are the most impactful and actionable, well-documented sustainability behaviors nutrition educators should be addressing in their consumer nutrition education efforts, the authors chose to conduct a narrative, rather than a systematic, review of the literature [36]. Narrative reviews employ a less-structured means of gathering and analyzing data and are more so guided by reviewer expertise [37], unlike systematic reviews, which aim to identify, assess, and synthesize literature in response to a more specific query, and use more explicit methods of data extraction and synthesis and more stringent reporting guidelines [37].

This narrative review was conducted between March and September 2021. Due to the broad scope of the research question, a variety of article types were included in the search, including original research, systematic reviews, more narrowly-defined narrative reviews, commentaries that were well-supported with references, and SD guidance documents, such as the 2015 U.S. Dietary Guidelines Scientific Advisory Committee Report [38], the EAT–*Lancet* Commission report [10], and the position paper on sustainability and dietary guidance from the Society for Nutrition Education and Behavior [23]. All literature outside of the aforementioned guidance was identified using Google Scholar, PubMED, and Web of Science, and had been published since 2010. The first search round used the key terms “sustainab *” and “sustainable diet *”; then, these terms were searched in combination with the terms “nutrition”, “nutrition education”, and “education” in a second-round search. The articles from the search were compiled in Microsoft Excel where notes delineated themes important for nutrition education. The articles were reviewed by the three authors and themes were discussed until 100% agreement was reached.

Based on inductive inference from articles collected from the first two rounds, as well as the authors’ experience as nutrition educators, six areas of further exploration were identified: reducing meat/plant-based diets, food waste, ultra-processed foods, local foods, fish and seafood, and conserving energy when cooking food. A third search round, conducted by a doctoral student who is also a registered dietitian nutritionist and nutrition educator (G.E.B.), combined the aforementioned terms with search terms related to the six further exploration areas, namely, “plant-based”, “meat”, “red meat”, “food waste”, “ultra-processed”, “local food”, “short food supply chain *”, “alternative food network *”, “seafood”, “cook *”, and “appliance”. These articles were compiled and analyzed in a similar fashion as the first two rounds.

Finally, since SD research is constantly being published, additional articles were reviewed as they came to the attention of the authors via press releases, food- and nutrition-related email list-servs (e.g., the Society for Nutrition Education and Behavior “SNEEZE” email list and the Community Food Security Coalition’s “COMFOOD” email list), and social media sites such as ResearchGate.

From this review, the authors reviewed the themes from the compiled literature and elicited five potentially impactful, evidence-based recommendations. The data are presented below, in the manner the authors believe may be the most to least impact. In Section 4, additional considerations for the recommendations’ inclusion in nutrition education practice are discussed.

### 3.1. Shift towards a More Plant-Based Diet

The most common and consistently made consumer SD recommendation was for consumers to shift to consumption of more plant-based foods in lieu of animal-based foods. This is due to the strong evidence that the production of animal-based foods—particularly beef and other ruminants, and to a lesser extent pork, poultry, eggs, and dairy— results in greater environmental impacts than the production of plant-based foods [17,39,40,41,42,43,44,45,46,47]. The environmental impacts of animal-based foods have been assessed using a variety of methodologies. Most commonly, these impacts have been evaluated via life cycle assessment of GHG emissions (i.e., a “cradle to grave” assessment of all emissions associated with the production of animal-based foods, including raw inputs and waste materials [48]), but also by examining land use, water use, and energy ratios (a ratio of how much energy is required to grow a food, versus how much energy the food provides in kilocalories or grams of protein). Notably, each method ranks animal-based foods as more detrimental to the environment than plant-based foods. Excessive environmental impacts attributable to the production of animal-based foods fall into three main categories [49]:

1. Upstream activities, including the production of feed crops for livestock, and the energy costs associated with constructing farm buildings and running equipment powered by fossil fuels;
2. Animal production activities, including nitrous oxide formation from animal waste, methane production from ruminant enteric fermentation, and the energy costs of maintaining livestock (e.g., heating and cooling);
3. Downstream activities, including energy costs associated with the transport, slaughter, processing, and packaging of livestock and related food products.

**Figure 1:**
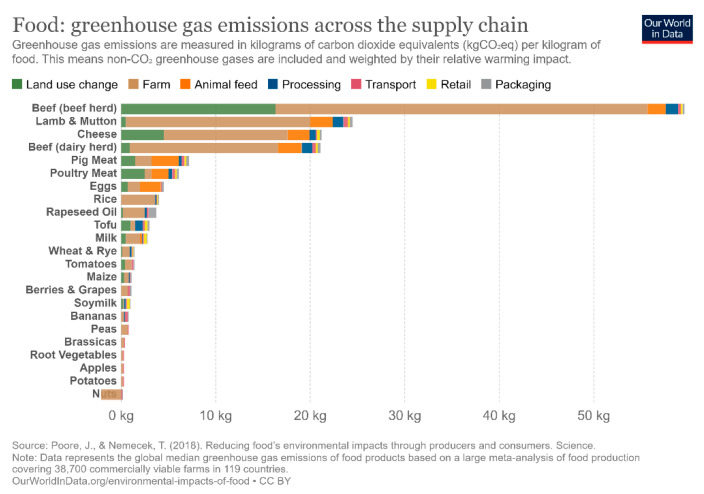
GHG emissions of select foods across the global supply chain. Reproduced by use of OurWorldInData’s CC-BY license, and with permission from Poole, the corresponding author of the original dataset.

When accounting for all sources of GHG emissions in the food chain, again, animal-based foods contribute considerably more carbon dioxide equivalents per kilogram than plant-based foods, and thus contribute more towards climate change, as shown in Figure 1, using data from Poore and Nemecek [50], and compiled using OurWorldInData [51].

The production of animal-based foods also uses a considerable amount of land and water. Livestock production uses 30% of the land on Earth [52], and has led to widespread deforestation and accelerated soil erosion [41,46]. Livestock also use copious amounts of water, due to the high volume of crops they eat, and one kilocalorie of beef is estimated to have been produced with 20 times more water than one kilocalorie of a grain food or starchy root vegetable [47]. Freshwater has been polluted by animal waste, antibiotics, hormones, and other industrial agriculture waste, making the livestock sector the biggest contributor to global water pollution [52].

Meat provides a poor return on investment for kilocalories provided in exchange for kilocalories consumed. For example, in comparison to the number of kilocalories consumed during their lifespans, the average chicken provides 12%, the average pork swine provides 10%, and the average beef cattle provides only 3% of these kilocalories back to their consumers [53]. With 36% of the world’s crop kilocalories being fed to livestock, and some countries, such as the US, feeding upwards of two-thirds of their crop kilocalories to livestock, it has been argued that the caloric efficiency of the world’s diet needs improvement to better feed humanity in the coming decades [53].

It is important to note that not all livestock, even of the same type, contribute similarly to environmental impact. For instance, 56% of GHG emissions from beef herds, as well as 61% of their land use, come from just 25% of the most environmentally-impactful producers [50]. However, no one agricultural practice was identified as being able to predict the environmental impact of foods on a global scale, due to the highly individualized nature of improving farming practices at each site [50]. For beef cattle specifically, Poore and Nemecek identified cases of both grass-fed and feedlot operations that had GHG emissions below the median, which had done so by prioritizing different types of environmentally-friendly farming practices [50]. From these observations, it may not be prudent for a nutrition educator to promote one type of livestock production method over another (e.g., “only buy grass-fed beef”), as such a generalization does not consider the nuance of such practices and their environmental effects.

A review of research regarding consumer attitudes towards plant-based diets showed that many consumers have shifted towards the consumption of more plant-based diets due to a host of objective and perceived benefits to their health and the environment [54]. Yet, others have not done so, with perceived barriers such as hedonistic attachments to meat consumption being cited [54,55,56]. Thus, opportunities to satiate consumers’ preference for meat, through more sustainable means, have been explored. An emerging market trend has been the substitution of conventional meat products with alternatives [57]. These alternatives fit into three main categories:

1. Plant-based foods that aim to mimic conventional meat in terms of taste and texture (often called plant-based meat alternatives, or meat analogs) [58,59,60];
2. Meat cultivated in a lab setting from muscle cells of conventional meat animals, (often called cultivated, cultured, or lab-grown meat) [61,62];
3. Meat from insects such as locusts (called insect meat) [63].

A comparison of pros and cons of each conventional meat alternative are listed in Table 1.

Meat alternatives have been gaining traction with consumers and becoming household names and have even permeated the restaurant market. For example, the international fast-food chain Burger King^®^, in collaboration with several manufacturers of plant-based meat, has introduced meatless versions of its Whopper^®^ burger in numerous countries since 2019, with plans to expand even further [67]; in response, McDonald’s^®^, in partnership with Beyond Meat^®^, rolled out consumer tests of its new “McPlant™” in 2021 in select US and European markets [68]. However, with concerns being raised about the ultra-processed quality, and high sodium content, of plant-based meat [61], nutrition educators should consider the promotion of plant-based diets to consumers in a way that limits the replacement of meat with plant-based meat. One such example may be to provide recipes utilizing less-processed plant proteins, such as legumes and nuts.

While vegan and vegetarian dietary patterns have been shown to produce less than half the GHG emissions of omnivorous dietary patterns [45], and use less land and water [41,45], these dietary patterns may put some at risk for nutrient deficiencies. As Magkos et al. have succinctly argued, several nutrients; including protein, iron, zinc, calcium, vitamin D, and vitamin B12, are more present or bioavailable in animal-based foods [69]. Additionally, iodine may also be a nutrient of concern for vegetarians and vegans in countries without adequate iodine supplementation in the food supply [70,71]. For vegetarians or vegans, or those who are considering such diets, nutrition educators should emphasize potential nutrient inadequacies and promote strategies to overcome them, such as pairing complementary proteins, facilitating nonheme iron absorption with vitamin C, and dietary supplementation when appropriate. For consumers not interested in giving up meat, providing so-called “flexitarian” strategies to reduce the amount of meat eaten can still reduce dietary environmental impacts [72], and may be the best combination of environmental stewardship and nutritional adequacy for many consumers [73].

With global meat intake having increased by >30% from 1961 to 2011, and even more so in emerging economies, such as Brazil and China [74], it is a very strong priority for work to be done to change consumer demand through the promotion of plant-based diets, which has the capacity to, consequently, incentivize the production of more plant-based foods and disincentivize the growth of meat production. These SD promotion needs, combined with the notable health benefits of a more plant-based diet and concerns associated with excessive meat intake (particularly red and processed meat) [75,76,77], lead the authors to conclude that this recommendation is the most important SD concept for nutrition educators to integrate into their work.

### 3.2. Mitigate Food Waste

“Food loss” and “food waste” both address edible food products that were intended for human consumption but were discarded or diverted elsewhere, and ultimately not ingested by people [78]. However, food loss refers to food discard/diversion pre-retail, and food waste refers to it at the retail or consumer level [78]. Food loss and food waste can happen at all steps of the food chain, from the field to the plate [79].

Estimates on the prevalence of food loss and food waste vary. In 2011, the FAO estimated that up to one-third of all food grown globally was lost or wasted [78], but due to concerns regarding the robustness of postharvest food loss and waste measures, some have challenged this estimate and offered more conservative predictions [80,81]. Regardless, the literature reflects wide agreement that food loss and food waste is a substantial issue, with estimates hovering around a billion tons of food lost or wasted, annually [82]. More recent approaches have been used to estimate food loss and food waste, separately, using different methodologies. In 2019, FAO estimated global food loss to be 14% of global food production [83], whereas a 2021 report by the United Nations Environment Programme estimated food waste at 17% [84]. Although both estimates lacked data from many countries, particularly LMIC [85], most higher-income nations were well represented in the data set, thus fitting the authors’ second assumption for this review.

**Figure 2:**
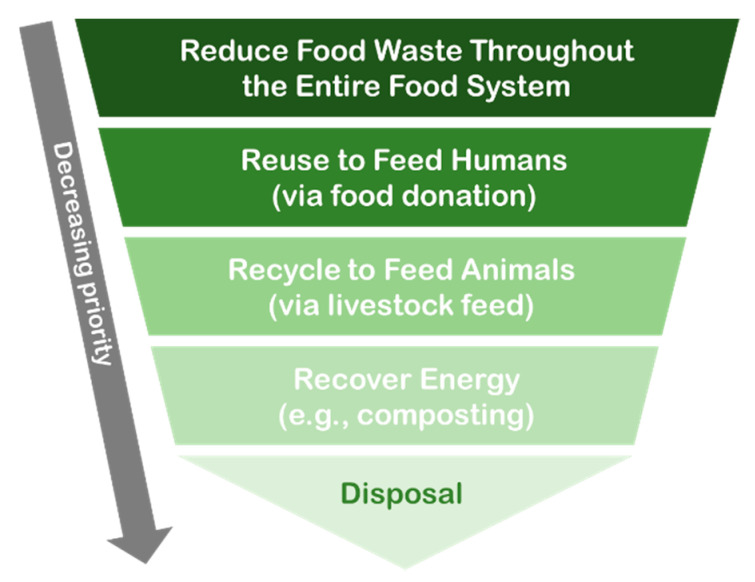
A simplified food waste hierarchy.

To assist in the management of food waste more effectively, food waste hierarchies, such as the one in Figure 2, have been developed [86,87,88]. Adapted from the waste hierarchy model first presented in Europe via the 1975 European Parliament Council Directive on Waste [86,87], the food waste hierarchy has been adopted by many governments as a model for the promotion of food waste mitigation. Notably, all adaptations are in agreement regarding the priority of the actions that can be taken prior to the inevitable need for some “disposal”, e.g., in landfills [89,90,91].

The first step in the hierarchy refers to placing the highest priority on the reduction of food waste throughout the entire food system. Of note, 61% of global food waste has been estimated to occur at the consumer level, totaling 569 million tons of food in 2019 [84], so this is a strong priority for nutrition education. Of the remaining recommendations to “re-use” food, particularly through donation to those experiencing food insecurity (e.g., food bank, food pantry, and soup kitchen contributions); “recycle” food waste to feed animals, namely livestock (e.g., using table scraps for pig feed); and “recover energy” (e.g., composting, anaerobic digestion, and industrial use), consumers can play a direct role in all but recycle. Thus, these steps constitute additional considerations regarding messages that can be communicated through nutrition education.

One such way that the mitigation of food waste can be promoted by nutrition educators is via “food resource management” (FRM), which has been defined by the US Department of Agriculture as the “increased ability to buy, grow, or otherwise appropriately obtain, prepare, and store food that meets nutritional needs [92]”. FRM behaviors have been established as mediators for both improved diet quality [93] and food security [94], and are an already established component of many nutrition education programs, particularly those funded by the US government. FRM behaviors identified as being particularly helpful for consumers to mitigate food waste include having a proper understanding of date labels on packaged foods (e.g., “use by”, “best by”, and “sell by” dates), utilizing leftovers, having more realistic expectations regarding food appearance (e.g., “ugly” produce), avoiding shopping practices such as buying in bulk when the extra food will not be eaten, and the proper food storage [95,96,97]. Interestingly, although excess food packaging is also a source of waste, the climate impact of wasted food is much higher than that of packaging [98]. Therefore, advising consumers to select products packaged in amounts that can be consumed before spoiling, which may result in more packaging, might be, as Aschemann-Witzel et al. called it, a “lesser evil” for nutrition educators to consider [96].

### 3.3. Limit Consumption of Ultra-Processed Foods

“Ultra-processed foods” (UPF), including soft drinks, sweets, instant or pre-prepared meals, processed meat items, prepackaged breads, and many other food items, are foods that have undergone a high degree of transformation through a series of chemical and industrial processes (e.g., hydrolysis, hydrogenation) and/or the addition of non-food ingredients (e.g., preservatives, emulsifiers, and food colorings) [99,100]. UPF are “designed to create highly profitable (low-cost ingredients, long shelf-life, emphatic branding), convenient (ready-to-consume), hyper-palatable products [99]”. UPF differ from other types of processed foods that have undergone processing steps such as refining, canning, or fermentation, but that have not been exposed to chemical alteration or the addition of many artificial ingredients [99]. While UPF are sometimes marketed as healthy, low-fat, low-sugar, organic, and/or vegan [101], most are high-energy, low nutrient-dense foods that are frequently over-consumed, likely due to their high palatability and the low level of satiety they typically provide due to their low protein and fiber content [101].

A 2020 review of UPF and food system sustainability associated UPF with “intensive agriculture” practices, such as increased deforestation and biodiversity loss; pollution from increased herbicides, pesticides, plastics, and worldwide distribution; economic hardship for small farmers in LMIC due to highly-subsidized, imported foods from higher-income nations; and the destruction of culinary traditions and social practices of sharing food [102]. Due to the variety of foods categorized under UPF and the relative newness of the term, it is difficult to determine the full contribution of UPF to dietary GHG emissions. In an attempt to quantify these comprehensive impacts, researchers created a model from Australian dietary data, which estimated that “lower quality” diets (i.e., those with high UPF consumption) had a 307% greater impact on GHG emissions than diets based on Australian dietary guidelines [103]. Another study of Australian dietary patterns found that discretionary foods, which have a “significant overlap” with UPF, account for approximately one-third of diet-related carbon dioxide equivalents, as well as more than one-third of water use (35%), land use (35%), and energy use (39%) from the Australian diet [104]. Tilman and Clark suggested that if the trend of high UPF consumption continues unchecked, these foods may contribute to an “estimated 80% increase in global agricultural GHG emissions from food production and to global land clearing” by 2050 [105].

Despite limited quantitative evidence in terms of UPF’s contributions to environmental sustainability, the literature leaves little room for doubt regarding UPF’s detrimental impact on nutritional adequacy and health. Researchers have long recognized the strength certain food product factors play in consumer food choice; such as palatability and taste, long shelf life, having little or no cooking or preparation time, the ability to be eaten anywhere, and being packaged in individual containers; particularly when such food products are of a low price and successfully branded [106,107]. Since UPF are manufactured to meet these aims, it is not surprising that cross-sectional evidence has indicated that UPF consumption is excessive in many countries, with high-income nations such as the United States, Canada, and the United Kingdom having estimated dietary caloric contributions from UPF being upward of 60% of all calories consumed [108,109,110]. Further, growing global UPF consumption [111] has even resulted in consumption rates around 30% in emerging economies such as Brazil, Mexico, and Chile [112,113,114].

UPF overconsumption has been linked to numerous chronic illnesses including obesity, cancer, type 2 diabetes, cardiovascular disease, and metabolic syndrome, without any positive health outcomes [115,116]. Evidence has suggested that this may be due to the inherent properties of UPF, such as higher glycemic glucose equivalent scores than lesser-processed and raw foods [117]. Moreover, in a study performed in a controlled environment, human subjects ate more and gained more weight on an ultra-processed diet than on a minimally processed diet, even when the two diets were matched for kilocalories, fiber, and macronutrients [118]. Given the strength of the evidence regarding the health issues associated with the overconsumption of UPF, along with the aforementioned sustainability concerns, education aimed at their reduction is a high priority area for nutrition education.

### 3.4. Engage in Local Food Systems

The local food movement, a counterculture effort to modify consumer’s heavy reliance on global, industrialized, food systems through increased consumer engagement in local food systems, can be traced back in the literature to Gussow and Clancy’s original premise for SD nutrition education back in 1986, where they urged consumers to “buy locally produced foods to support regional agriculture that preserves farmland and is less energy intensive [27]”. However, interest in shifting towards a diet more focused on local foods only truly gained momentum in the 21st century and is now being given its due among many countries across the globe [119,120,121]. Although no universal definition exists, local food systems, also called “alternative food networks” or “short food supply chains”, typically refer to food that is produced within a “close proximity” of where it is eventually sold, whether that is within a few miles or across state or province lines [122]. Some countries have established official definitions for local food, each of them varying widely [119]. For instance, the US has defined local food as that which is grown within 644 km (400 miles) from where it is sold [123], and France has defined it as food grown within 150 km (93 miles) [124]. Canada has defined local food as food sold within 50 km (31 miles) from the border of the province or territory in which it originated [125]. Due to Canada’s size, this becomes a distance that can be, in total, over 1000 km (621 miles) from farm to point of sale [119]. In practice, defining what constitutes local food in a particular community has been negotiated, contested, and re-negotiated among various food system actors, including growers, distributers, policymakers, and consumers [126].

It has been difficult to establish the true environmental impact of local food systems’ use versus the use of food that has been received via a “global” or “long” food supply chain, especially since there are many different types of local food systems, and some food chains even include both “local” and “global” aspects [127]. Studies that investigated the energy costs of local and global food systems have found that global food systems typically outperform their local counterparts, in terms of total energy use and GHG emissions [127,128,129]. These studies have concluded that differences in scale have allowed global food systems to be more efficient with regards to energy use, since global food systems can increase efficiency by transporting more product at the same time, thus using less travel energy per unit of product [128,129], and using food technology to speed up certain steps in food processing, such as cheese maturation, to save energy overall [128]. Furthermore, critics argue that local food systems rely on more individual consumer transport, as consumers have been predicted to drive farther and more out of their way to purchase locally-sourced food, which creates a higher reliance on fossil fuels [129].

This, as some have noted [119,128], is counterintuitive to what is known as the “food miles” concept, which was coined by UK activists in the 1990s to describe what had been thought of as the lower inherent GHG emissions of food grown locally [130]. The food miles concept is complicated by the fact that certain transportation types, such as air freight, have been shown to produce more GHG emissions over short distances than other modes of travel have generated over longer distances [131].

Notably, whether or not GHG emissions are lessened more via long or short supply chains, food transportation has been estimated to account for only 10–15% of GHG emissions in the agricultural sector [132]. A smaller subset of literature has suggested that it is possible for local food systems to have a lower environmental impact by changing food system operations, such as creating shorter transportation distances to retail sites for food, employees, and consumers, or using less energy for coolers, warehouses, and point of sales (e.g., farm stores and farm stands) [133]. When these variables were compared in 10 total cases of local and global food systems (five local, four long-chain, and one mixed), there was wide variation, even among cases with the same distribution type (e.g., supermarkets); neither local nor global food systems demonstrated greater overall energy savings, and the most energy-efficient cases saved energy costs in different aspects of the distribution system [133]. In a more direct comparison, Plawecki et al. compared the carbon footprints of a local, organic leaf lettuce grown in Michigan versus that associated with a conventional leaf lettuce grown in California [134]. Because the Michigan lettuce was able to be grown in a hoop house which did not need external heating, the inputs needed were much less than the inputs needed for the California lettuce (e.g., irrigation, refrigeration, transportation), and the California lettuces’ emissions, when brought to Michigan, resulted in a footprint that was over 4 times greater [134].

Aside from GHG emissions or energy use considerations, research has suggested that local food systems contribute to sustainability in a multitude of other ways. For example, a review of 90 case studies of best practices of European local food providers indicated that local food systems have positively impacted economic, environmental, and social aspects of sustainability [135]. Local food systems have outperformed global food systems in important sustainability metrics, namely biodiversity, animal welfare, governance, and resiliency [127,128]. Also, when locally-produced foods were ranked against more global foods of the same type (e.g., a local, regional French wine versus a similar French generic table wine produced for export) on a multitude of sustainability attributes, the local foods consistently outranked their global counterparts [128]. Local food systems have also been shown to stimulate local economies by providing employment and generating profit [136,137,138,139]; provide growers with a heightened sense of civic engagement towards their communities [140]; improve access to fruits and vegetables, especially in low-income areas where access may be otherwise scarce [141,142,143,144,145]; and improve health outcomes for the community, including reduced body mass index, better eating behaviors, and greater reported overall health [146,147,148,149].

Another consideration for local food systems and their sustainability is resiliency. Local food systems have been theorized and demonstrated to more effectively respond and adapt to crisis situations, including economic changes [150,151], climate change [152,153], and pandemics (such as the ongoing, novel coronavirus COVID-19 outbreak) [154,155,156,157,158], whereas global food supply chains have faltered under similar pressures. As severe weather events have been predicted to increase globally over the next decades [9], and the true risk of future economic crises or pandemics is unknown, the relevancy of food system resiliency to crises will likely persist in the near future, which further supports the notion that actively engaging in local food systems should have a place in nutrition education.

### 3.5. Choose Sustainable Seafood

Seafood (which mainly refers to fish and shellfish, but can also refer to other edible marine life, like seaweed) is a nutritionally important food group containing several essential nutrients that are difficult to obtain from other food sources, such as vitamin D, iodine, selenium, and the omega-3 fatty acids: eicosapentaenoic acid and docosahexaenoic acid [159]. A nutrient analysis published in 2021 by Golden et al. named the top seven nutrient-rich animal-based foods as being small and large pelagic fish, bivalves, aquatic mammals, salmonids, carps, and cephalopods [160]; all seafood! Some food-based dietary guidelines, such as the US Dietary Guidelines for Americans, have recommended seafood consumption at least twice weekly for good health [161]. Yet, despite the health benefits of seafood, its industry has been rife with sustainability issues.

Overfishing, a practice of wild catching that does not ensure the maintenance of a species’ wild populations, has steadily increased despite FAO surveillance and policy action efforts [162]. In 2017, 34.2% of all marine fish stocks were considered overfished, which was over 3 times greater than 1974 estimates [162]. Those who overfish have been found to engage in many other unscrupulous wild catching practices, including importing of illegal or unregulated seafood [163,164], renaming and mislabeling of seafood that goes to market [165], and accidental bycatch of non-seafood marine life, such as turtles and dolphins, affecting ocean ecosystems [166,167].

Aquaculture, or “farmed” seafood, has been estimated to represent almost half of the global seafood trade [162]. The modern aquaculture movement began in the second half of the 20th century, as fish farming technology increased, and concerns of depleting wild fish stocks were raised [168]. Whereas the volume of wild fishing has remained relatively constant since the 1980s, global aquaculture production has exploded in the same time frame [169], such that the volume of seafood produced via aquaculture production is now on par with wild catching [162]. The widespread expansion and industrialization of the aquaculture industry continued to create a host of environmental issues, including loss of aquatic habitats and agricultural land, water pollution from aquaculture feed and waste, and the disruption of ecosystems including biodiversity loss [170].

However, over the last decade, improved practices for sustainable aquaculture have been proposed [171,172,173,174], and a 2021 review remarked that aquaculture practices had, indeed, become more sustainable from 1997 to 2017, particularly with regards to fish feed efficiency and nutrition [175]. In fact, practices have improved to the extent that organizations such as the Monterey Bay Aquarium Seafood Watch, which creates science-based recommendations to help consumers make ocean-friendly choices, now recommends some responsibly farmed seafood species, such as artic char, scallops, and oysters, as better choices than their wild counterparts [176]. Generally speaking, of all aquaculture species, farmed filter-feeder bivalves and seaweed have shown promise for greatly benefitting ecosystems, due to their abilities to purify water and absorb carbon, respectively [175].

It has been suggested that products identified as “sustainable seafood” should be evaluated based on the species, country of origin, and production practices [177]. Regarding species, generally, seafood that is low-trophic, or low on the food chain, has been deemed more sustainable [178]. For instance, studies that have categorized seafood species on a nutrition-sustainability matrix have recognized small pelagic fish, such as herring and mackerel, as being both highly nutritious and highly sustainable [179,180]. Country of origin has been used as a proxy [181] to determine if the seafood product may have been produced via unethical practices, such as “Illegal, Unreported and Unregulated” fishing [182,183,184]. Production practices can refer to wild versus aquaculture seafood, as for some species there have been differences in sustainability between the two production types [185,186], or it can refer to the existence of specific fishing or aquaculture practices that can influence seafood sustainability, such as using gill nets [187] or giving aquaculture animals fishmeal as feed [188,189]. Overall, the identification of sustainable seafood has been recognized as a complex process [186], that has left even nutrition professionals [190] confused with regards to how to identify sustainable seafood or find reliable means of identifying them [191].

Sustainability labels, or eco-labels, have been used on some wild and aquaculture seafood products to simplify sustainable seafood identification for consumers [192,193]. Eco-labels consist of both on-the-package labeling (e.g., the Marine Stewardship Council certifications for sustainable wild-caught seafood and the Aquaculture Stewardship Council certifications for sustainable aquaculture seafood), or rating systems that do not appear on the package, which are accessed using online or print materials (e.g., the Seafood Watch by the Monterey Bay Aquarium in California, US). Although most of these eco-labels have been managed by third-party organizations [192], some governments, such as in France and Thailand, have created their own sustainable seafood certification systems [193]. On-the-package labels denote that the fishery or farm which produced the product complied with all standards of the certifying organization. Rating systems, on the other hand, do not certify individual producers, but they instead designate products to consume or avoid, based on criteria such as species, farming method, country of origin, body of water, or other package certifications. When available, consumers can use both types of eco-labels to make sustainable seafood purchasing decisions; however, research has suggested that consumers may not have the knowledge or familiarity with eco-labels to properly use them [194]. Thus, due to the nutritional benefits of seafood, critical sustainability concerns, and consumers’ lack of knowledge regarding the identification of sustainable seafood, even when seafood eco-labels are available, this is yet another priority area for nutrition educators to consider regarding the integration of SD messages in their educational endeavors.

## 4. Discussion

### 4.1. Summary and Rationale for Specific Recommendations Offered

Based on a broad review of consumer practices that support sustainable food systems, the SD recommendations to (1) shift towards a more plant-based diet; (2) mitigate food waste; (3) limit consumption of ultra-processed foods; (4) engage in local food systems; and (5) choose sustainable seafood were identified as priority areas for nutrition educators. These recommendations emerged, in the descending order presented, based on the strength of the evidence regarding their potential for positively impacting the food system’s sustainability and their role in maintaining SD. Table 2 outlines examples of how nutrition educators may consider incorporating the five recommendations into nutrition education programming, with an emphasis on adult audiences; however, please note that this is not a comprehensive list.

Many of the nutrition education topics provided in Table 2 are currently taught in community nutrition education programs; for example, FRM is a required education component of the US Expanded Food and Nutrition Education Program [92]. However, in the authors’ experiences, many educators and low-income participants are completely unaware that food system sustainability is even an issue. As such, nutrition educators should be made aware of this, and should incorporate these recommendations with messaging to combine both nutrition and sustainability education. Continuing with our example, FRM skills are not only able to help participants stretch food dollars and improve food security, but they are critical skills to help reduce food waste. Consumers who may be less motivated to practice FRM skills to save money or cook healthy meals may be motivated by learning about the sheer amount of food wasted, creating an opportunity for behavior change that would not be reached via nutrition-focused education alone.

Despite each of the recommendations above being presented independently, they are complementary, and should be combined during nutrition education when applicable. For example, when promoting plant-based diets, nutrition educators should motivate consumers to choose less-processed plant proteins, such as legumes, nuts, and seeds, as opposed to plant-based meat, which is considered a UPF. As consumers are encouraged to purchase less-processed foods, local foods from farmers’ markets and community-supported agriculture, and more sustainable seafood, such as pelagic fish, they may need additional culinary and FRM education to ensure that these more sustainable behaviors do not lead to increased consumer costs and food waste.

Since projections suggest there is little doubt that freshwater shortages are imminent, and sustainable management of water systems are called for to prevent drought and scarcity [199,200,201], the authors sought to ensure this issue was addressed. However, no direct consumer food-related actions were evident. In fact, the literature suggests that the actions via which consumers have the greatest potential for reducing water shortages are to adopt a more plant-based diet [10], reduce intake of UPFs [104], and choose sustainable seafood [170]. As such, and in light of the fact that the 2019 “Guiding Principles for Sustainable Healthy Diets” from the Food and Agriculture Organization of the United Nations and World Health Organization recommend that “safe and clean drinking water (be consumed) as the fluid of choice” [202]; no additional recommendations were made.

One of the areas of further exploration considered by the authors during the literature search that was not included in the final SD recommendations for nutrition educators was energy conservation in food preparation. While it has been argued that “enhancing the use of more efficient and safer cooking systems among households is…an issue of vital importance [203]”, studies have shown that, overall, cooking constitutes a small fraction of the energy use and GHG emissions associated with human food consumption, as compared to the impacts associated with agricultural practices, food processing, and food waste [204,205]. In some cases wherein foods with relatively low environmental impacts, specifically fruits and vegetables, are cooked, the home cooking process constitutes a substantial portion of the products’ total life cycle environmental impacts [206,207,208]; yet, with global fruit and vegetable intake being far below what has been recommended for SD [10], it is the authors’ belief that using higher-energy methods to increase palatability (e.g., roasting vegetables), is a necessary trade-off nutrition educators should not be afraid to make in order to promote SD. Additionally, estimates regarding the environmental impact of cooking foods varies widely, depending on the degree to which modern, clean energy appliances are used [208,209], and the impacts of particular activities associated with cooking (e.g., refrigerated vs. dry food storage and hand washing dishes vs. the use of a dishwasher) appear to remain too complex to interpret.

The literature suggests that the environmental impact of preparing food using basic, fresh ingredients has a lower environmental impact than purchasing ready-made meals [210], and that sustainable cooking methods favor the use of microwave ovens [206,210,211], pressure cooking [183], steaming [183], electric grilling [206,210], and the use of appliances specifically engineered to efficiently cook one specific food, such as pasta or rice [212,213], as opposed to the use of high-heat, conventional cooking methods. However, the purchase and replacement of these multiple appliances may lead to additional electronics waste, which has an undetermined impact on the environment. Again, considering the relatively small environmental impact of household food preparation, coupled with the aforementioned complications that do not support strong, actionable recommendations for nutrition educators, at this time the authors do not believe this is an area for which any strong SD recommendations can be made.

### 4.2. Integrating Sustainable Diet Information in Nutrition Education

The following section provides an example of incorporating the SD recommendations into a nutrition education curriculum based on the authors’ experiences. The authors (G.E.B. and D.M.P.K.) both work, in part, for the *Expanded Food and Nutrition Education Program* (EFNEP). EFNEP is a community-based nutrition education program funded by the US Department of Agriculture, National Institute of Food and Agriculture, that is administered via partnerships with 76 land grant universities from all 50 US states, the District of Columbia, and 6 US territories [188]. Established in 1968, EFNEP’s aim is to improve the nutrition-related behaviors of low-income US families through nutrition education [188]. A paraprofessional education model is employed in EFNEP, i.e., the frontline educators are “indigenous” to the low-income communities in which they teach, and they do not have formal college training in nutrition [189]. By leveraging local community partnerships, EFNEP paraprofessional educators provide evidence-based nutrition education interventions to low-income parents of young children, low-income pregnant and breastfeeding women, and low-income youth from kindergarten to the 12th grade [188].

The authors’ first foray into SD incorporation into EFNEP programming was, admittedly, not for the purposes of improving sustainability, but instead was tested as a potential means of enticing low-income, older adolescents to increase their vegetable intake. The authors were working on a new curriculum for which increasing vegetable intake was a primary aim, but the original lessons, based on improved health and even managing weight, developed as they had proven to be ineffective. After some brainstorming among the development team, and further review of the factors that drive behavior change among adolescents, the authors deduced that since adolescents have been posited to be more likely to make dietary changes for social justice reasons than health reasons alone, this type of educational approach may improve the curriculum’s effectiveness [214]. The concept that climate change may be of particular interest to teens had recently been reinforced in the news via youth climate activist Greta Thunberg’s ”School Strike for Climate”, and later research suggesting that the residuals of her activism effected youth and adults alike [215,216].

A lesson was created wherein the teens were required to watch a short video on sustainability that focused on decreasing meat consumption and, instead, increasing the intake of less environmentally burdensome products, such as chicken and pork, but primarily vegetables. After watching the video, the adolescents were split into teams and competed for points based on their responses to questions posed that reinforced the concepts taught in the video. In subsequent lessons, the original activities that were based on improving health and managing weight through increased vegetable consumption were still taught, both through game-based approaches (i.e., gamification).

Qualitative assessment based on interviews with a sub-sample of participating teens from two high schools, conducted by a doctoral student working on the team, revealed that of the 10 lessons in the curriculum, the SD lesson was ranked among the top 2 [217]. Further, its impact was successful beyond what had been hoped for. In addition to the lesson having a profound impact among most of the teens interviewed in terms of reducing red meat consumption and increasing their vegetable intake, the teens provided qualitative evidence that they had shared what they learned at home, and many indicated their families were cooking at home more, rather than going out for “burgers”, and that their parents were preparing more plant-based diets [217]. 

Nutrition educators should take heed of an additional “lesson learned” shortly thereafter. After the SD lesson was conducted at another teaching location, one of the teens’ classroom teachers wrote a rather angry email to the local program supervisor suggesting that the program was trying to turn the students into vegetarians, based on evidence that lacked a scientific base. Of course, this had not been our aim, and the video encouraged “decreased” red meat consumption, not vegetarianism—which was never mentioned—but still, one take-home point was valid. Given our need to do concept-testing on this varied approach to increased vegetable consumption, as previously noted, we had used a video that had been created by an SD advocacy group. While the overall content of the video was well-aligned with current SD recommendations, upon further examination we found that the statistics provided were not verifiable. Nutrition educators who intend to incorporate SD education into their work should learn from our oversight and be cautious to make sure all the information they share on what remains a contentious topic to many (i.e., those strongly attached to a meat-centered diet) is supported in the literature.

**Figure 3:**
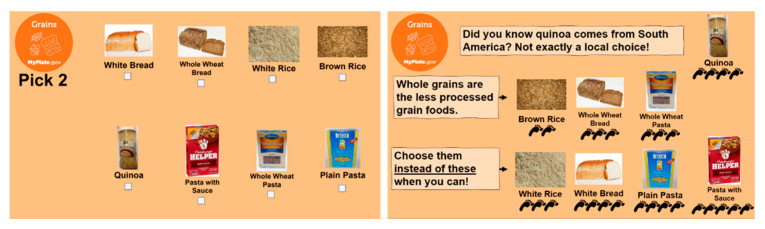
Sample materials from “Supermarket Sweep for Sustainability”. On the left is an example of the grocery items from the grains group from the lesson’s online version. On the right, the “carbon footprints” of the grain items are revealed on the lesson’s slide presentation. All lesson materials are available at https://www.efnephelps.org/revitup (accessed on 30 September 2021).

However, this incident served to fuel our interest in this area and precipitate the investigation on which this manuscript is based. It also prompted our development of a new, thoroughly research-based replacement lesson that includes content on all five SD recommendations. The replacement lesson, titled “Supermarket Sweep for Sustainability”, is loosely based off the game show Supermarket Sweep. First, teens learn about SD and strategies to shop for more sustainable foods: (1) “Use the Power of Plants!”, (2) “Eat in the Here and Now!” (i.e., choose local and seasonal foods), and (3) “Skip the Processed Junk!” (i.e., UPFs). Then, in teams, the teens choose grocery items to fill their “shopping baskets” with based on the SD strategies they learned. Eventually, the educator reveals the number of “carbon footprints” each food produces (Figure 3). The team with the fewest total number of “carbon footprints”, i.e., the most sustainable shopping basket, wins the game. Throughout the lesson, sustainable seafood eco-labels and reducing food waste are also explained as other ways to eat sustainably. Although no assessment data have been developed, to date, pilot lessons have indicated that this lesson is equally well-received by adolescents.

In addition to providing training to the EFNEP paraprofessional educators such that they could successfully teach the SD lesson to older adolescents, we provided a more comprehensive 2-day (6 h) food system sustainability training, followed by a brainstorming session aimed at determining (1) if they believed SD concepts should be integrated into EFNEP adult programming; (2) based on what they had learned, what they believed would be of greatest value to their program participants; and (3) what aspects of what they had learned would be the most actionable for their clientele and most easily be integrated into what they taught. The paraprofessional educators, who again, hailed from the low-income, minority populations they taught, were shocked by the presentation. They made it clear that they felt disenfranchised by the fact that no one had ever before discussed with them the fragility of the food system, and the impact it may have on their families’ food and water supply in the future. They were zealous in their commitment about carrying the message forward to everyone they knew, including their class participants. While they recognized that their class participants’ incomes limited their abilities to purchase pricier options (e.g., some seafood with sustainability certifications), they strongly believed that if their participants’ awareness regarding SD was developed, it would support the paraprofessionals’ work, as they taught participants aspects of food resource management (i.e., the management of financial commitments allocated to food), that could avoid food waste through more extensive food shopping, preparation, and storage planning efforts. They also suggested that it would lend added support to their educational efforts regarding the adoption of more plant-based diets, reduced intake of red meat, increased vegetable intake, increased frequency of cooking at home, and reduced consumption of processed snacks and beverages.

None of the paraprofessional educators had previously heard of community-supported agriculture (CSA; i.e., a system wherein individuals enter into some type of mutually supportive relationship with a farmer(s) in exchange for fresh, locally-grown, seasonal foods). When instructed, as part of the training, to do an internet search of CSA opportunities within the counties in which they worked, some found opportunities for individuals to spend time working at a farm in exchange for food (rather than offering financial support), which they believed might be of interest to some of their adult participants and their families. Additionally, the paraprofessional educators also believed that print-outs of information regarding locations and hours of operation for local farm stands and farmers’ markets, and of the Monterey Bay Aquarium’s wallet-sized Seafood Watch National Consumer Guide [176], should be made available to their participants, many of whom they believed would be interested in using them. While the results of this training have not been assessed, and the aforementioned actions have not yet been formally incorporated into our adult EFNEP lessons, it was clear that the paraprofessional staff wanted to spread the word about SD.

The above examples of how SD concepts can be incorporated into nutrition education are limited to the authors’ experiences, and should be coupled with the shared experiences of others who have developed audience-specific nutrition education inclusive of information regarding SD. For example, curricula like “In Defense of Food [218]” can spark additional ideas among readers that will translate into effective means for incorporating SD information into a variety of nutrition education venues for various audiences.

## Figures and Tables

**Table 1:** Pros and cons of three main types of alternatives to conventional meat.

Type of Alternative to Conventional Meat	Pros	Cons
Plant-based meat	Variety of products (e.g., burgers, sausages, chicken, and seafood mimetics) with increasing market share [61,62]	Ultra-processed [61], high in sodium [61,62], and wide ranges in nutrient profiles among products [62]
Cultured meat	Identical taste, texture, and nutrient profile to conventional meat [64]	Energy-intensive, expensive production costs, and yet to be scaled to meet mass demand [64]
Insect meat	Nutritious and low environmental impact [63]	Potential food allergen [65], disgusting/unappetizing to some [66]

**Table 2:** Examples of how the 5 Sustainable Diet Recommendations can be incorporated into nutrition education programming.

Sustainable Diet Recommendation	Ways to Incorporate Recommendations into Programming
Shift towards a more plant-based diet	Demonstrate and provide plant-based recipes, and in addition to their health benefits, use food system sustainability as an additional means of promoting the consumption of fruits, vegetables, whole grains, nuts, seeds, and legumes
2. Mitigate food waste	Provide data regarding the amount of food wasted (approximately 40% in the US [195]), highlight how money can be saved when food waste is reduced, and teach FRM skills such as meal planning, proper food storage, and how to interpret date labels on foods, with a focus on reducing food waste
3. Limit consumption of ultra-processed foods	Provide culinary education and encourage cooking homemade meals, promote easy snacks and meals that use less-processed foods, and provide information regarding UPF and their impact on both human health and food system sustainability
4. Engage in local food systems	Provide addresses of local farmers’ markets, farm stands, and community-supported agriculture (CSA); provide lists of local and seasonal foods; and if working with low-income participants, share information on CSA work programs [196] and incentive programs at local farmers’ markets, and if needed, work with vendors to provide incentives for low-income community members (e.g., doubled value of benefit dollars [197,198])
5. Choose sustainable seafood	Provide information on seafood sustainability issues such as overfishing, teach how to interpret seafood eco-labels, and provide information on independent seafood consumer guides
